# Using Bacteria to Determine Protein Kinase Specificity and Predict Target Substrates

**DOI:** 10.1371/journal.pone.0052747

**Published:** 2012-12-26

**Authors:** Michael F. Chou, Sladjana Prisic, Joshua M. Lubner, George M. Church, Robert N. Husson, Daniel Schwartz

**Affiliations:** 1 Department of Genetics, Harvard Medical School, Boston, Massachusetts, United States of America; 2 Division of Infectious Diseases, Children’s Hospital Boston and Harvard Medical School, Boston, Massachusetts, United States of America; 3 Department of Physiology and Neurobiology, University of Connecticut, Storrs, Connecticut, United States of America; University of Toronto, Canada

## Abstract

The identification of protein kinase targets remains a significant bottleneck for our understanding of signal transduction in normal and diseased cellular states. Kinases recognize their substrates in part through sequence motifs on substrate proteins, which, to date, have most effectively been elucidated using combinatorial peptide library approaches. Here, we present and demonstrate the ProPeL method for easy and accurate discovery of kinase specificity motifs through the use of native bacterial proteomes that serve as *in vivo* libraries for thousands of simultaneous phosphorylation reactions. Using recombinant kinases expressed in *E. coli* followed by mass spectrometry, the approach accurately recapitulated the well-established motif preferences of human basophilic (Protein Kinase A) and acidophilic (Casein Kinase II) kinases. These motifs, derived for PKA and CK II using only bacterial sequence data, were then further validated by utilizing them in conjunction with the *scan-x* software program to computationally predict known human phosphorylation sites with high confidence.

## Introduction

Protein phosphorylation provides one of the primary means of transducing cellular signals, and as such has been utilized by a majority of organisms that span all domains of life [1]. Extensive research has been carried out to uncover the existence and specific location of phosphorylation sites on proteins as a means of understanding protein function and regulation. Although advances in enrichment and detection technologies have led to an exponential increase in known phosphorylation sites on substrate proteins over the past decade [2], an important limitation of these strategies is that they do not provide information on the kinases responsible for the phosphorylation events. The absence of kinase-specific information thus greatly limits our ability to understand the role of individual kinases within dynamic signal transduction networks.

Many variables contribute to the likelihood of a kinase targeting a given protein in the cell including i) temporal expression of the kinase and substrate, ii) subcellular localization of the kinase and substrate, iii) physical interactions between the kinase, substrate and often other proteins, and iv) the existence of sequence specificity determinants (also known as motifs) on the substrate protein. Given that kinase specificity motifs can vary widely (compare, for example, the RxRxxS sequence preference of Akt kinase [3] to the YMxM sequence preference of the Insulin Receptor kinase [4]), it is not surprising that they have served as a major means of generating hypotheses regarding kinase/substrate pairs that can then be experimentally verified. Thus, kinase specificity motifs have been of significant importance in elucidating kinase function and cellular signaling mechanisms.

To date, the most established and widely used methods for kinase specificity determination have involved incubation of purified recombinant kinase with combinatorial peptide libraries *in vitro* [5], [6]. Depending on the format of the reaction (i.e., in solution or on streptavidin-coated membranes), read-out of the specificity is accomplished by either Edman degradation or autoradiography. At present, it is not practical to use tandem mass spectrometry in conjunction with combinatorial peptide library methods because, among other reasons, it would require *de novo* peptide sequencing by mass spectrometry, which is currently challenging. Although they have provided valuable data for many kinases, combinatorial peptide library based methods share several limitations (Table 1).

**Table 1:** Comparison of combinatorial peptide library screening, proteome-derived library, and ProPeL motif discovery methodologies.

	Combinatorial peptide libraryscreening	Proteome-derived libraries	ProPeL
Need for purified recombinant kinase?	Yes	Yes	**No**
Use of radioactivity in some assays?	Yes	**No**	**No**
Can use tandem MS to determinesequence?	No	**Yes**	**Yes**
Can accurately determine disfavoredresidues at motif positions?	No	**Yes**	**Yes**
Can determine specificity of all residuesat all motif positions?	No*	**Yes**	**Yes**
Motif width limit?	Yes (limited by library construction)	Dependent on protocol**	**No**
Demonstrated successfulphosphorylation predictionsfrom resulting motifs?	**Yes (Scansite)**	No	**Yes (** ***scan-x*** **)**
Assessment of kinase specificity under*in vivo* conditions?	No	No	**Yes**
Up front costs?	Higher	**Lower**	**Lower**
Potential for incompletedephosphorylation of endogenousphosphoproteins?	**Not applicable**	Yes	**Not applicable**
Relative number of protocol steps(i.e., ease of protocol).	Most	Fewer	**Fewest**

* Phosphorylatable residues (Ser and Thr) and Cys are not included in combinatorial peptide libraries used for kinase specificity determination.

** In proteome-derived libraries motif width limits depend on whether the kinase reaction is performed before or after proteolytic peptide digestion.

Most recently, several groups have expanded upon an approach first presented by Huang et al. in 2007 [7] to use phosphatase treated intact proteins from eukaryotic cellular lysate as a “proteome-derived” peptide library for subsequent *in vitro* kinase reactions. This approach has been used to both query for potential kinase substrates *in vitro* and to derive kinase motifs [8], [9], [10]. While these methods have the substantial advantage of being able to use tandem mass spectrometry as a peptide readout, they suffer from the need for large amounts of purified active recombinant kinase, the potential for incomplete dephosphorylation of endogenous phosphoproteins, and the assessment of phosphorylation under often unfavorable *in vitro* conditions.

Here we address the aforementioned deficiencies of both the combinatorial and “proteome-based” approaches to kinase specificity motif determination by presenting a novel and simple strategy that we call ProPeL (which stands for Proteomic Peptide Library). Importantly, the ProPeL methodology represents the first use of a live bacterium (here, *E. coli*) acting as an *in vivo* peptide library for thousands of simultaneous phosphorylation reactions carried out by an exogenous kinase.

Briefly, a kinase of interest is cloned and expressed in *E. coli* using standard techniques. Following induction, the active kinase (which uses cellular ATP as a cofactor) can phosphorylate the native *E. coli* proteome in a manner that is consistent with the sequence specificity of the kinase. Typically, such modifications to the host proteome would go unmeasured, however, in our approach they serve as a convenient readout of the kinase motif. In order to detect these phosphorylation sites, the bacteria are lysed, proteins are digested using trypsin, phosphopeptides are enriched using SCX/IMAC [11], and the resulting phosphopeptides are sequenced by tandem mass spectrometry. Despite the fact that the differentially phosphorylated *E. coli* peptides are not natural substrates for the expressed kinase, phosphorylation motifs are statistically identified using the *motif-x* [12] and pLogo [13] software with *E. coli* selected as a background database to account for the proteomic environment within which the reaction occurs.

Importantly, the ProPeL strategy is made possible by the fact that *E. coli*, i) lacks any eukaryotic-like serine/threonine kinases, ii) has only two kinases with known serine/threonine activity, and iii) has very low levels of endogenous serine and threonine phosphorylation [14]. A comparison of the different methods for determining kinase specificity is provided in Table 1.

## Results

As a proof of principle, we applied the ProPeL approach to two human kinases, Protein Kinase A (PKA) and Casein Kinase II (CK II), both of which have well-defined motifs [6], [15]. In total, the methodology resulted in the detection of 806 phosphorylation sites in *E. coli* expressing PKA, and 467 phosphorylation sites in *E. coli* expressing CK II. By comparison, negative controls (untransformed *E. coli* and *E. coli* expressing empty plasmid) led to the identification of only 23 endogenous phosphorylation sites, consistent with the known low background phosphorylation levels in *E. coli* [14] (see Table S1). Following removal of known endogenous phosphorylation sites obtained from both negative controls in the present study and an additional study of *E. coli* phosphorylation [14], 794 PKA phosphorylation sites and 458 CK II phosphorylation sites remained, which served as the data sets for motif analyses.

**Figure 1:**
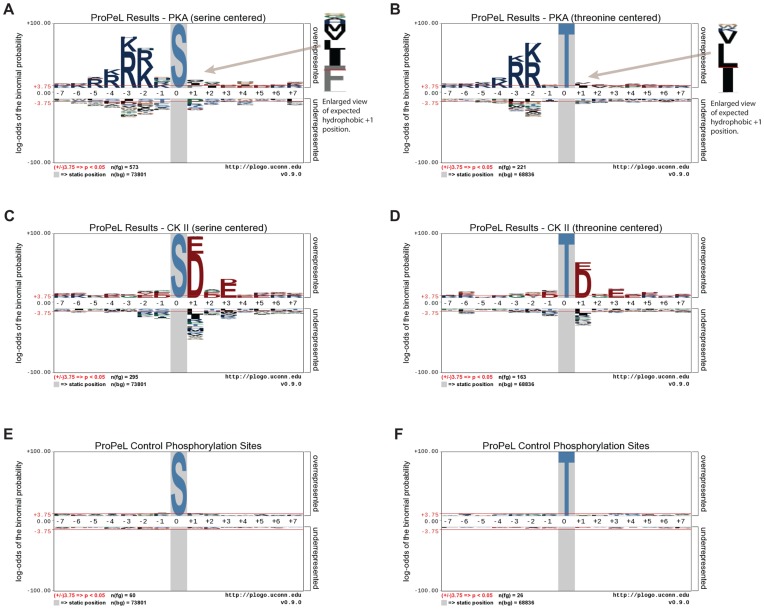
pLogo representations of substrate sequence specificities. pLogos for Protein Kinase A (A, B), Casein Kinase II (C, D), and control (E, F) illustrate preferred residues by position. Note, pLogos are derived from phosphorylation sites in *E. coli* obtained using the ProPeL methodology (after subtraction of endogenous phosphorylation sites). In each pLogo, residue heights are proportional to their log binomial probabilities in the context of the *E. coli* background with residues above the x-axis indicating overrepresentation and residues below the x-axis indicating underrepresentation. The central residue in each pLogo is fixed and denotes the modification site. The pLogos and corresponding extracted motifs (see Figure 2) are highly consistent with the known basophilic specificity of PKA and acidophilic specificity of CK II. Additionally, the control phosphorylation sites (i.e., endogenous *E. coli* phosphorylation sites) do not conform to a motif and lack any statistically significant residues.

**Figure 2:**
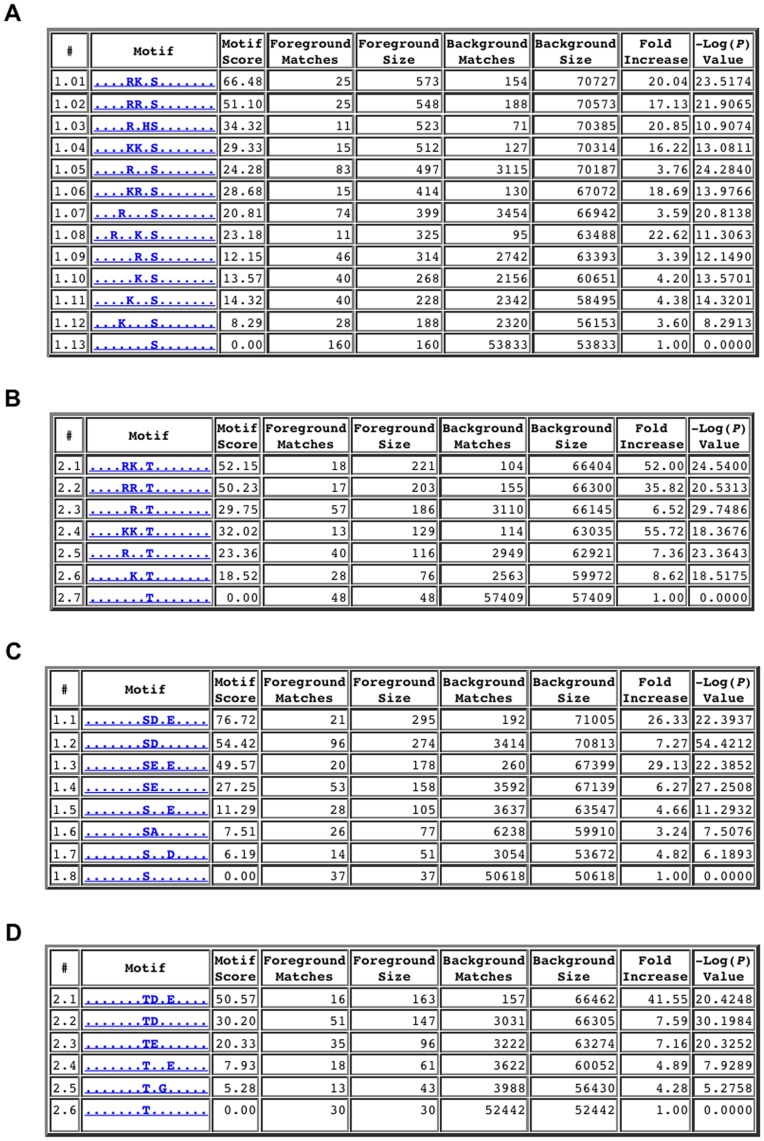
*motif-x* analyses for PKA (A and B) and CK II (C and D). These motif extraction results illustrate the inter-residue correlations found among the phosphorylated peptides identified using the ProPeL methodology, and are highly consistent with the previously established consensus sequences for the PKA and CK II kinases.

In both cases, the motif determined using the ProPeL methodology mirrored the established kinase consensus sequences (Figure 1A–D). Specifically, the most prominent previously characterized specificity determinants of PKA – a preference for basic residues upstream of the modification site at the −2 and −3 positions as well as a hydrophobic residue preference at the +1 position [6] – were clearly evident in the serine- and threonine-centered pLogos for PKA (Figures 1A and 1B respectively). Similarly, the most critical specificity determinants of CK II phosphorylation – a preference for acidic residues upstream and downstream of the phosphorylation site, with the +1 and +3 positions being most important [15] – were also clearly evident in the serine- and threonine-centered pLogos for CK II (Figures 1C and 1D respectively). It should be noted that the y-axes of the pLogos shown in Figure 1 are on a logarithmic scale. Thus, for example, while the R at the −3 position in Figure 1A has an associated *p*-value of 10^−55^, the hydrophobic cluster (I/L/M/V/F) at the +1 position, albeit smaller, still has a highly significant *p*-value of 10^−10^. Motif deconvolution using the *motif-x* algorithm [12] further corroborated the pLogo results, yielding motifs highly consistent with the known specificities of PKA and CK II (see Figure 2). By comparison, pLogos for known endogenous *E. coli* phosphorylation sites (i.e., 86 sites from our negative controls and from the Macek et al. study [14]) revealed no statistically significant residues, and thus no overall motif (Figure 1E and 1F). Finally, comparison of the phosphorylation sites obtained in the PKA and CK II experiments revealed only negligible overlap (21 peptides out of over 1200 total peptides), with the majority of overlapping peptides bearing similarity to both the PKA and CK II consensus sequences. As such, it is highly unlikely that expression of PKA and CK II resulted in the activation of native *E. coli* kinases.

**Figure 3:**
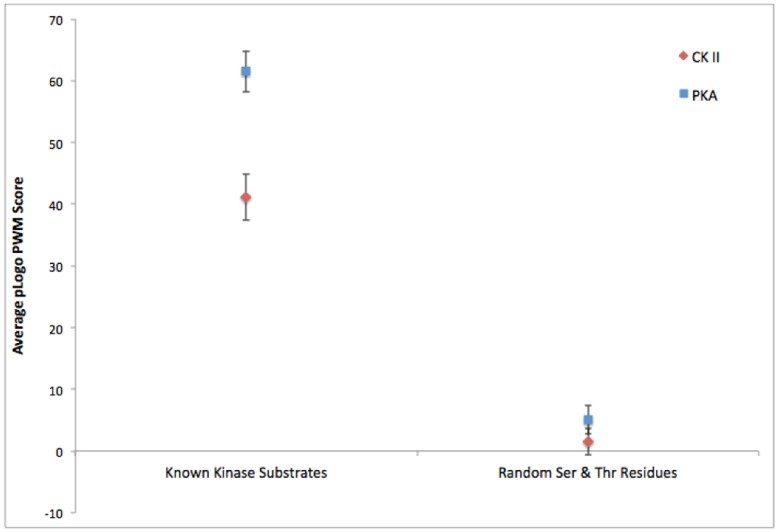
Goodness-of-fit of the pLogos derived from ProPeL and actual known kinase substrates versus random substrates. Average pLogo position weight matrix scores of CK II (red) and PKA (blue) pLogos when scanned against known human substrates from the PhosphoSitePlus database compared to average scores obtained from scanning CK II and PKA pLogos against an equivalent number of random human serine and threonine residues. Error bars represent 95% confidence intervals.

To quantitatively assess the accuracy of the motifs obtained through the ProPeL method, we used a previously devised scoring strategy, the *scan-x* score [13], that utilizes the raw pLogo position weight matrix values to measure the goodness-of-fit between a pLogo and a particular sequence of interest (see Figure 3). Scoring 320 known human PKA substrates retrieved from the PhosphoSitePlus [16] database with the PKA pLogo obtained using the ProPeL method, and an equivalent number of random phosphorylatable residues from the human proteome with the same PKA pLogo, yielded a highly statistically significant difference in average *scan-x* score (61.5 versus 5.1, Mann-Whitney U = 94323.0, *n* = 320, *p*<10^−75^). Similarly, scoring 348 known human CK II substrates with the CK II pLogo, and an equivalent number of random human phosphorylatable residues with the same CK II pLogo, also yielded a highly statistically significant difference in average *scan-x* score (41.1 versus 1.5, Mann-Whitney U = 104230.5, *n* = 348, *p*<10^−60^). These results both demonstrate that the pLogos obtained via the ProPeL methodology can be used to accurately discern the difference between a random serine or threonine residue and a true PKA or CK II phosphorylation site, and in turn that the pLogos are a strong representation of known PKA and CK II specificities.

We then used *scan-x* to identify potential PKA and CK II native kinase targets in the human proteome using these same pLogos (Tables 2 and 3). In the case of PKA, the top 100 predicted phosphorylation sites (out of nearly 1.17 million potentially phosphorylatable unique serine- and threonine-centered 15 mers in the human proteome) contained two sites (on proteins KCNH2 and SOX9) known to be phosphorylated by PKA (data not shown, hypergeometric *p*-value <10^−3^). Within just the top 20 predictions (Table 2), eight sites were previously verified to be phosphorylated (by an unknown kinase) *in vivo* (hypergeometric *p*-value <10^−5^), and 4 proteins had associations with PKA either directly or through protein family members. In the case of CK II (Table 3), the 3^rd^ highest scoring site in the entire human proteome is, in fact, a *known* CK II substrate (MCM2, Ser139, *scan-x* score = 118.1). In addition, the highest scoring candidate CK II substrate in the human proteome (NADAP, Ser312, *scan-x* score = 123.2) has also been shown to be phosphorylated at our predicted site (Ser 312) by 27 independent tandem mass spectrometry studies [16], and was most recently shown to interact with CK II [17] suggesting that it is a likely CK II substrate. Overall, of the top 20 CK II predictions, 30% (6/20) of sites are already known to be phosphorylated by CK II at the precise predicted site, and 70% (14/20) have known, experimentally verified CK II interactions. Note, that the probability of selecting even a single known CK II phosphorylation site by chance is extremely low ∼348/1,170,000 (or 0.03%), thus finding 6 out of 20 known CK II sites has a hypergeometric *p*-value of <10^−17^. Given the limited present knowledge of the phosphorylation state of proteins, it is also striking that 80% (16/20) of the top 20 predicted CK II phosphorylation sites were previously shown to be phosphorylated (hypergeometric *p*-value <10^−13^); most, in dozens of independent experiments. The remaining 4 of the top 20 predicted CK II phosphorylation sites had no prior experimental evidence of phosphorylation. However, these 4 predictions are all contained within tryptic peptides that are longer than 35 amino acids, and are thus also unlikely to be detected using standard high-throughput tandem mass spectrometry workflows.

**Table 2:** Top 20 *scan-x* PKA phosphorylation predictions based on a human whole proteome scan with the PKA motif obtained using the ProPeL methodology.

*scan-x* rank*	UniProt ID	Site	Known phosphorylation site? (if yes, in howmany experiments has it been reported?**)	Known PKA association?
1	KCNH7_HUMAN	S896	Yes (7 experiments)	No, but family member KCNH2 is phosphorylated by PKA. [34]
2	FLII_HUMAN	S436	Yes (163 experiments)	No
3	SEM4G_HUMAN	S713	Yes (1 experiment)	No
4	CHD8_HUMAN	S506	No***	Yes, shown to bind PKA. [35]
5	ADML_HUMAN	S153	No***	No
6	H2AFB_HUMAN	S10	No***	No
7	KCNK5_HUMAN	S266	No	No, but family members KCNK2, KCNK3, and KCNK9 are phosphorylated by PKA. [36], [37]
8	ATAD2_HUMAN	S379	Yes (6 experiments)	No
9	MCLN2_HUMAN	S530	No***	No, but family member Mucolipin 1 is phosphorylated by PKA. [38]
10	FOXD1_HUMAN	S58	No***	No
11	RBM34_HUMAN	S14	Yes (81 experiments)	No
12	PTPRG_HUMAN	S55	No	No
13	GLTL1_HUMAN	S520	No	No
14	PHF14_HUMAN	S835	Yes (54 experiments)	No
15	KIRR1_HUMAN	S527	No***	No
16	UBP51_HUMAN	S356	Yes (1 experiment)	No
17	EI24_HUMAN	S46	Yes (49 experiments)	No
18	RED2_HUMAN	S30	No***	No
19	DYSF_HUMAN	S593	No	No
20	TRI17_HUMAN	S59	No***	No

* Out of 1,168,144 total serine and threonine residues.

** From the PhosphoSitePlus database.

*** Tryptic peptide containing the predicted phosphorylation site less than length 10 or greater than length 35.

**Table 3:** Top 20 *scan-x* CK II phosphorylation predictions based on a human whole proteome scan with the CK II motif obtained using the ProPeL methodology.

*scan-x* rank*	UniProt ID	Site	Known phosphorylation site? (if yes, in howmany experiments has it been reported?**)	Known CK II association?
1	NADAP_HUMAN	S312	Yes (27 experiments)	Yes [17]
2	DAXX_HUMAN	S443	No***	Yes, CK II phosphorylates at alternate sites. [39]
3	MCM2_HUMAN	S139	Yes (235 experiments)	Yes, CK II phosphorylates at predicted site. [40]
4	RPC7_HUMAN	S157	Yes (4 experiments)	No
5	BAZ1B_HUMAN	S1259	No***	No
6	TFP11_HUMAN	S98	Yes (26 experiments)	No
7	BMS1_HUMAN	S442	No***	Yes [17]
8	ENPL_HUMAN	S306	Yes (14 experiments)	Yes [41]
9	NUCL_HUMAN	S145	Yes (80 experiments)	Yes [42]
10	CCD97_HUMAN	S257	Yes (3 experiments)	No
11	GLRPX_HUMAN	S205	Yes (29 experiments)	Yes [17]
12	HS90A_HUMAN	S231	Yes (45 experiments)	Yes, CK II phosphorylates at predicted site. [43]
13	HS90B_HUMAN	S226	Yes (101 experiments)	Yes, CK II phosphorylates at predicted site. [43]
14	CENPB_HUMAN	S456	Yes (1 experiment)	Yes, CK II phosphorylates at predicted site. [44]
15	PRPF3_HUMAN	S619	Yes (23 experiments)	Yes, CK II phosphorylates at alternate site. [45]
16	CCD94_HUMAN	S211	Yes (20 experiments)	No
17	MYH9_HUMAN	S1943	Yes (764 experiments)	Yes, CK II phosphorylates at predicted site. [46]
18	F123B_HUMAN	S928	No***	No
19	SIAL_HUMAN	S149	Yes**** (3 experiments)	Yes, CK II phosphorylates at predicted site. [47]
20	TOP2A_HUMAN	S1106	Yes (18 experiments)	Yes, CK II phosphorylates at alternate sites. [48], [49]

* Out of 1,168,144 total serine and threonine residues.

** From the PhosphoSitePlus database.

*** Tryptic peptide containing the predicted phosphorylation site greater than 35 residues in length.

**** Phosphorylated at homologous site in rat and cow.

The aforementioned results demonstrate that the motifs obtained via the ProPeL methodology can be used to scan whole proteomes in order to predict new high-confidence phosphorylation sites specific to a given kinase. Therefore, in addition to uncovering the motifs for kinases with unknown sequence specificities, by using a bacterial expression system, the ProPeL methodology can be used in conjunction with *scan-x* as an efficient tool to predict kinase substrates within their native proteomes.

**Figure 4:**
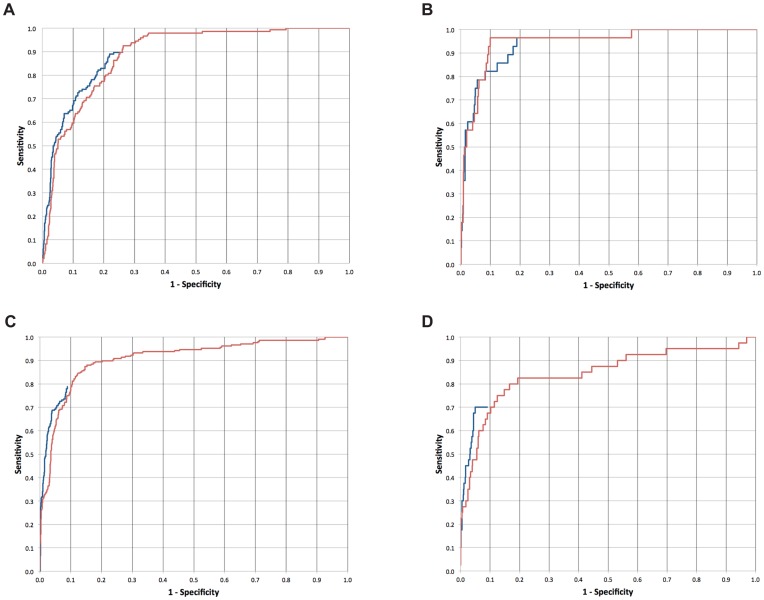
Receiver Operating Characteristic (ROC) curves for the *scan-x* and Scansite PKA and CK II kinase specific predictors. These curves illustrate the tradeoff between sensitivity and specificity achieved by the ProPeL based *scan-x* (red) and combinatorial peptide library based Scansite (blue) predictors, and indicate the similarity of results achieved using these experimentally orthogonal approaches. Panels (A) and (B) are based on PKA serine and threonine predictions, respectively, while panels (C) and (D) are based on CK II serine and threonine predictions, respectively. The Scansite web server does not score all phosphorylatable residues in a given sequence, which results in partial ROC curves.

Finally, to assess the tradeoff between the sensitivity and specificity of ProPeL-based *scan-x* predictions, and to compare these results to those obtained using the combinatorial peptide library based Scansite predictor [18], we generated “gold-standard” positive and negative kinase data sets for PKA and CK II based on known data contained within the PhosphoSitePlus database. These data were then used to create receiver operating characteristic (ROC) curves for both the *scan-x* and Scansite PKA and CK II specific predictors (see Figure 4A–D). Aside from illustrating the strong predictive capacity of the ProPeL/*scan-x* methodology, the ROC curves also provide evidence that no significant predictive biases arise from using bacterially derived peptide libraries to make eukaryotic predictions and that the scoring matrices derived from synthetic peptides and bacteria are virtually interchangeable. It is worth noting that many of the known substrates in the “gold-standard” that we used were determined after the release of Scansite in 2003. Inspection of a subsample of research articles demonstrating PKA and CK II phosphorylation sites contained within the PhosphoSitePlus database revealed that a significant number of sites were in fact experimentally verified as a direct result of performing Scansite analyses [19], [20]. Thus, the Scansite ROC curves in Figures 4A–D likely represent a slight overestimation of Scansite predictive capacity.

## Discussion

The present study represents the first application of an exogenous *in vivo* system being used as a reaction vessel to query the specificity of a single protein kinase. The success of the methodology is largely due to the ease of expressing kinases in *E. coli* – an organism with a sufficiently large proteome to provide a complex substrate library and extremely low endogenous phosphorylation levels [14]. As such, each phosphorylation event detected in a ProPeL experiment is likely to be the result of the expressed exogenous kinase, which is unencumbered by the background noise of other interfering kinases (Figure 1). Additionally, motifs discovered with this strategy are the result of analyzing many substrates to discover a statistically significant pattern [12]. Thus, the identity of the individual protein targets, and whether they originate from human or *E. coli* cells is irrelevant to the task of determining a motif for the kinase. This premise is strongly supported by our experimental results in which motif construction from *E. coli* proteins phosphorylated by CK II and PKA led to the direct prediction of a large number of previously validated phosphorylation sites and known CK II and PKA substrates in the human proteome (Tables 2 and 3).

Aside from its simplicity, the ProPeL approach has a number of other benefits over the well-established combinatorial peptide library approaches and the recent proteome-derived library approaches (Table 1). Most significantly, by shifting the kinase reaction into *E. coli*, there is an immediate benefit of having the enzyme perform its function in a more physiologically relevant biochemical environment, thus allowing kinases as well as their cognate bacterial substrates to assume structured conformations, and obviating the need to adjust a variety of experimental parameters (buffer conditions, ATP concentrations, etc.) or to purify large quantities of intact and active recombinant kinases. Additionally, when compared to the most current combinatorial peptide library approaches [5], there is an economic benefit because the initial cost of a ProPeL experiment is a fraction of the cost of combinatorial peptide library synthesis. We anticipate that this cost advantage will only be magnified as tandem mass spectrometry core facilities and instrumentation become more accessible and strategies for phosphopeptide enrichment become more efficient.

In the present study, large portions of the ProPeL methodology were carried out independently by two different laboratories (one lab for each kinase), using slightly varying interpretations of the SCX/IMAC protocol (see Methods section for details). The fact that these independent experiments yielded hundreds of phosphorylation sites, both of which accurately recapitulated the expected motifs, provides evidence for the robustness of the approach. Additionally, the use of acidophilic (Casein Kinase II) and basophilic (Protein Kinase A) kinases from opposite sides of the kinase phylogeny suggests a potential for applicability to intermediate kinases without significant bias from the *E. coli* intracellular environment.

While the human proteome is much larger than that of *E. coli*, we demonstrate here that the diversity of the *E. coli* proteome is more than sufficient for the discovery of protein kinase motifs, which typically contain no more than two to three major specificity-determining positions [21]. In fact, through motif deconvolution using *motif-x* we were able to detect numerous motifs with 3 fixed positions (Figure 2), thus indicating that the *E. coli* proteome is large and diverse enough to detect inter-residue correlations. Nevertheless, it should be possible to use the ProPeL method with alternate bacterial expressions systems (such as members of the *Bacillus* genus) as a way to interrogate different sequence repertoires available for motif determination.

It is well established that expressing a variety of exogenous proteins in *E. coli*, including kinases, may result in a range of toxicities to the bacterial host. Because of this, as with other protein expression systems, some kinases will likely require some fine-tuning of expression and culture conditions. Additionally, there exist kinases that require specific ligand binding (calcium/calmodulin [22], AMP [23], etc.) or phosphorylation events by upstream kinases for activity [24], and others that require a priming phosphorylation event for substrate specificity [25]. In some cases it will be possible to circumvent these issues by expressing only kinase catalytic domains/subunits, or by co-expressing the given kinase with its necessary activating (or priming) enzyme/ligand; however, in some cases the ProPeL methodology may prove unsuitable. Although we do not expect the ProPeL methodology to be effective for all kinases, even if 20% of kinases could be effectively expressed in bacteria, this would represent over one hundred kinases in *Homo sapiens* alone whose sequence specificity could be determined rapidly and accurately. As an alternative, however, we propose here that one may also use the ProPeL approach *in vitro*, whereby active recombinant kinase is added directly to *E. coli* proteomic lysate (followed by the same enrichment and MS/MS steps described). In this way, recombinant kinases may be expressed, purified, and activated in any appropriate system, while still realizing the benefits of the low background phosphorylation levels in the *E. coli* proteome.

We also wish to note that while compiling this manuscript we became aware of work by the Huber lab at the University of Illinois demonstrating the use of bacterial phosphorylation to determine the specificity of several plant receptor-like kinases (RLKs). Their successful results (simultaneously under review at the time of this manuscript’s submission) further suggest that the methodology is broadly applicable and can be used to query the specificity of kinases from a wide range of species – the overwhelming majority of which remain yet unknown.

Even in this post-genomic era, elucidation of cellular interactions remains a significant bottleneck for our understanding of molecular mechanisms in humans and other species. ProPeL can rapidly decode the sequence-based determinants of kinase specificity and provide experimentalists with the first line of hypothesis generation necessary to fully annotate the breadth of kinase-substrate interactions. In turn, we can better understand the role of protein phosphorylation and how it affects cell physiology in health and disease.

## Methods

### ProPeL PKA Methods

#### Plasmids, strains and in vivo proteome phosphorylation

Human his-tagged full-length PRKACA/pBEV construct was provided by Vertex Pharmaceuticals Inc [26]. *Escherichia coli* OverExpress C41(DE3) cells from Lucigen were transformed and plated on Luria-Bertani (LB) plates supplemented with 50 µg/mL carbenicillin, with an untransformed control. Colonies were inoculated in LB broth supplemented with 50 µg/mL carbenicillin and grown up overnight at 37°C with shaking at 200 rpm. Overnight cultures were diluted 10 fold into fresh media and grown under the same conditions until OD600 reached 1, at which point protein expression was induced with 1 mM Isopropyl-β-D-1-thiogalactopyranoside (IPTG) and grown overnight. Cultures were centrifuged at 3000 g for 20 minutes and cell pellets were stored at −80°C until lysis.

#### Lysis and analysis of in vivo phosphorylation

Cell lysate was prepared according to Villén and Gygi [11] with minor modifications. Cell pellets from 50 mL cultures were resuspended in 3.3 mL lysis buffer (8M urea, 75 mM NaCl, 60 mM Tris, pH 8.2) supplemented with 2 Complete Mini protease inhibitor tablets (Roche) per 10 mL and Phosphatase Inhibitor Cocktail 1 (Calbiochem). Cells were lysed by sonication using 4×30 second pulses at 100 Watts (Sonic Dismembrator 60, Fisher Scientific), with rest on ice between pulses. Crude lysate was clarified by centrifugation at 20000 g and 4°C for 10 minutes. Protein concentrations were determined by Bradford Protein Assay (Bio-Rad) and phosphorylation level was evaluated by SDS-PAGE with Pro-Q Diamond Phosphoprotein stain (Life Technologies), with total protein evaluated by GelCode Blue staining (Pierce). Lysates were stored at −80°C until further processing.

#### Protein digestion and phosphopeptide enrichment

Phosphopeptide enrichment was performed according to Villén and Gygi. Fifteen mg of each protein sample was reduced, alkylated and digested with trypsin. Peptides were desalted with 500 mg 3 cc tC18 SepPak Vac solid-phase extraction cartridges (Waters) and dried in a SpeedVac. Samples were fractionated by HPLC using a Resource S column (GE Healthcare) with 8 fractions collected according to Macek et al. [27], dried in a SpeedVac to remove acetonitrile, and desalted using 100 mg 1 cc SepPaks (Waters). Phosphopeptide enrichment was performed with PhosSelect iron affinity gel (Sigma) and desalted with StageTips made from C18 material (Proxeon), using the combined IMAC/StageTip method detailed in Villén and Gygi. Samples were dried down by vacuum centrifugation and stored at −20°C until mass spectrometry.

### ProPeL CK II Methods

#### Plasmids, strains and In vivo proteome phosphorylation

A plasmid containing the human CSNK2A1 gene in an Invitrogen Gateway donor vector (pDONR223) was kindly provided by The Broad Institute and was transferred using the standard Gateway protocol to the pDEST17 backbone for bacterial expression (Life Technologies). Escherichia coli OverExpress C41(DE3) cells (Lucigen) were transformed and plated on Luria-Bertani (LB) plates supplemented with 100 µg/mL ampicillin, with the empty vector pUC19 (New England Biolabs) serving as a control. Colonies were inoculated in LB broth supplemented with 100 µg/mL ampicillin and grown up overnight at 37°C with shaking at 250 rpm. Overnight cultures were diluted 50 fold into fresh media and grown under the same conditions until OD600 reached 0.6–0.7, at which point protein expression was induced with 1 mM Isopropyl-β-D-1-thiogalactopyranoside (IPTG) and grown for a further 3 hours. Cultures were pelleted by centrifugation at 3000 g and 4°C for 10 minutes. Pellets were stored at −70°C until lysis.

#### Lysis and analysis of in vivo phosphorylation

Cell lysate was prepared according to Villén and Gygi [11] with minor modifications. Cell pellets were resuspended in lysis buffer (8M urea, 75 mM NaCl, 60 mM Tris, pH 8.2) supplemented with Halt Protease Inhibitor Cocktail (EDTA-free) and Halt Phosphatase Inhibitor Cocktail (Pierce). Cells were lysed by sonication with a Fisher Sonic Dismembrator F60 at 15% power using 15–20 second pulses, with 1 minute rest on ice between pulses, until lysate was clear. Crude lysate was clarified by centrifugation at 2500 g and 4°C for 10 minutes. Protein concentrations were determined by Bichinchoninic Acid (BCA) Assay (Pierce) and phosphorylation level was evaluated by SDS-PAGE with Pro-Q Diamond Phosphoprotein stain (Life Technologies), with total protein evaluated by GelCode Blue staining (Pierce). Lysates were stored at −70°C until further processing.

#### Protein digestion and phosphopeptide enrichment

Phosphopeptide enrichment was performed according to Villén and Gygi. Samples were reduced, alkylated and digested with trypsin. Peptides were desalted with 500 mg 3 cc tC18 SepPak Vac solid-phase extraction cartridges (Waters), snap-frozen in liquid nitrogen and lyophilized. Samples were fractionated by HPLC using a polySULFOETHYL A SCX column (PolyLC) with 12 fractions collected, snap frozen in liquid nitrogen, partially lyophilized to remove acetonitrile, and desalted using 100 mg 1 cc SepPaks (Waters). Phosphopeptide enrichment was performed with PhosSelect iron affinity gel (Sigma) and desalted with StageTips made from C18 material (3M), as described in Rappsilber et al. [28] using the combined IMAC/StageTip method detailed in Villén and Gygi. Samples were dried down by vacuum centrifugation and stored at −20°C until mass spectrometry.

### Mass Spectrometry and Database Searching

For each of PKA and a negative control (untransformed cells), 8 SCX fractions were collected and enriched with IMAC as described above.

For each of CK II and a negative control (pUC19 alone), 12 SCX fractions were collected and enriched with IMAC as described above.

Before analysis with mass spectrometry, each fraction (either from a set of 8 or a set of 12) was dried down and resuspended in 30 uL of loading Buffer A (see below). Fractions 5, 6, 7 and 8 for a given starting sample were combined, dried and then resuspended to 30 uL loading buffer. For the samples with 12 fractions, fractions 9, 10, 11 and 12 were similarly combined before mass spectrometry was performed. For each fraction (or combined fractions), 4 uL was loaded (using buffer A) onto a C18 nanocapillary column with a pulled tip that sprayed directly into the inlet of a Thermo Fisher Scientific LTQ Orbitrap XL mass spectrometer.

Peptides were eluted from the column into the inlet of the mass spectrometer using an Agilent 1200 HPLC binary pump with a gradient that changed solvents from 100% to 65% Buffer A (0% to 35% Buffer B) over a 48 minute time period (Buffer A = 3% ACN, 0.125% formic acid in water, and Buffer B = 0.125% formic acid in acetonitrile).

During the course of the injection, the mass spectrometer was run using a TOP10 method (MS scan followed by Collision Induced Dissociation MS/MS on the top 10 most intense MS spectral peaks).

Each fraction’s spectra were searched using SEQUEST [29] against the *E. coli* proteome which included decoy database entries [30] and allowed for differential serine and threonine phosphate modifications (+79.966331), a differential methionine oxidation modification (15.9949146221) and a constant cysteine modification of +57.02146374.

Following SEQUEST analysis, peptides from spectra containing predicted serine and threonine phosphorylated peptides were summarized for motif analysis. In order to minimize false positives, for each of the two classes of peptide charges z = +2 and z = +3 and greater, minimum XCORR thresholds were chosen to be above the value of the highest XCORR for a decoy hit from the database. The deltaXCORR (the difference between the first and second hits to the databases) was always required to be 0.08. This corresponded to a predicted False Discovery Rate (FDR) of 0%, however it was still subject to statistical variations and may have included some small contamination of false positives.

For each input sample, peptides from each fraction (or combined fractions) were identified with a predicted FDR of 0% as described, and then the peptides were combined into a single list of non-redundant peptides for each fraction. Redundant peptides occurring across fractions, but not across samples were highly specific for a particular kinase, and many peptides were identified in more than one independent spectrum. Negative controls occasionally shared peptides with positive samples and with other negative controls.

The final lists of peptides used for each kinases’ motif analysis consisted of phosphopeptides that were not contained in controls nor previously reported to be found in the normal *E. coli* proteome [14].

### pLogo/*motif-x*/*scan-x* Analyses

#### pLogo generation

pLogo images were generated using in-house software. Specifically, pLogos depict residues proportional to the log-odds of their binomial probabilities with respect to a given background [13], [31], [32]. Here, the foreground data was obtained by mapping phosphorylated tryptic peptides (identified by MS/MS) back onto the E. coli proteome to retrieve necessary adjacent sequence information and to create an aligned data set of unique 15 mers centered on phosphorylation sites (using the same procedure as in motif-x analyses). The E. coli background data set was generated through alignment of all unique serine- or threonine-centered 15 mers in the E. coli proteome. In a pLogo, the most statistically significant residues appear closest to the x-axis, with residues above the x-axis indicating overrepresentation and those below the x-axis indicating underrepresentation. Fixed positions within the pLogo (e.g., the central position) are depicted on a grey background, and red horizontal lines denote the p<0.05 significance threshold (after Bonferroni correction). A detailed manuscript describing pLogos as well as a pLogo generation web site (http://plogo.uconn.edu) are currently in preparation.

#### motif-x analyses

motif-x analyses for both the PKA and CK II MS/MS peptide identification results were carried out using an internal version of the motif-x web tool [33] with the following parameters selected: central residue = S* or T*, width = 15, foreground occurrence threshold = 5, significance threshold = 0.00001, background database = NCBI E. coli proteome, and background central residue = S or T.

#### scan-x analyses of known and random kinase substrates

*scan-x* analyses of known and random substrates were carried out using an internal version of the *scan-x* software (described in detail in reference [13]). Known verified human substrates of PKA and CK II were retrieved from the PhosphoSitePlus database [16] (http://phosphosite.org), while random substrates were obtained by randomly choosing an equivalent number of serine/threonine 15 mers from the human proteome. A whole proteome scan was also carried out using the PKA and CK II pLogos against the entire SwissProt Human proteome containing nearly 1.17 million serine- and threonine-centered 15 mers to generate a ranked list of the highest scoring predicted substrates for those respective kinases.

#### ROC curve analysis

To obtain a “gold-standard” data set for ROC curve generation all human phosphorylation sites within the PhosphoSitePlus database which were only known to be phosphorylated by a *single* human kinase were retrieved. Sites shown to be phosphorylated by PKA were called “positive PKA sites” while those known to be phosphorylated by a different kinase were called “negative PKA sites”. Similarly, sites shown to be phosphorylated by CK II were called “positive CK II sites” while all other kinase specific phosphorylation sites were called “negative CK II sites”. *scan-x* analyses were carried out using an internal version of the *scan-x* software (described in detail in reference [13]. Scansite [18] analyses were carried out using the Scansite 3.0 web server (http://scansite3.mit.edu) with either the CK II or PKA matrices selected under minimum stringency setting (to retrieve a maximal amount of scoring data). To plot the ROC curves, sensitivity and specificity were calculated as previously described [13]. Because Scansite does not provide scoring results for every phosphorylatable residue provided in the input, ROC curves could not be drawn completely to the upper right hand corner (i.e., the point with 100% sensitivity and 100% false positive rate).

## Supporting Information

Table 1 **Raw mass spectrometry phosphorylated peptide sequence results for the Protein Kinase A (PKA), Casein Kinase II (CK II), and control ProPeL experiments.**
(XLS)
